# DNA Fingerprinting: Use of Autosomal Short Tandem Repeats in Forensic DNA Typing

**DOI:** 10.7759/cureus.30210

**Published:** 2022-10-12

**Authors:** Akshunna Keerti, Sudhir Ninave

**Affiliations:** 1 Medicine and Surgery, Jawaharlal Nehru Medical College, Datta Meghe Institute of Medical Sciences, Wardha, IND; 2 Forensic Medicine, Jawaharlal Nehru Medical College, Datta Meghe Institute of Medical Sciences, Wardha, IND

**Keywords:** restriction fragment length polymorphism, dna probe, dna database, str typing, forensic dna typing

## Abstract

Short tandem repeat (STR) markers for autosomal STR are used in forensic deoxyribonucleic acid (DNA) typing to track down the missing, verify family connections, and potentially connect suspects to crime sites. It is well acknowledged that forensically relevant genetic markers cannot predict phenotype. There is no evidence to support the claim that directly using forensic STR variations causes or indicates illness. Such an example would have significant ethical and permissible repercussions. It is essential to check the necessity to alert a blood donor or if a medical problem is identified during routine sample analysis. In this study, we assess the likelihood that forensic STRs might offer details beyond those required for primary identification. However, as the role of non-coding STRs in gene regulation is understood, the probability of discovering meaningful links is rising. For this review, Google Scholar, ScienceDirect, PubMed, and Google Search were all used to conduct a thorough electronic literature search. If they linked to the topic, thoughts, retrospective studies, observational studies, and first publications were considered. The case studies presented here highlight the critical role forensic DNA typing plays in reducing criminal risk and delivering conclusive evidence in cases. The primary method for forensic DNA typing is short tandem repeat (STR) typing. This discussion on the importance of STR markers to the criminal justice system is part of the present study. As unflinching proof of false beliefs and invaluable connections to the genuine culprits, DNA typing offers proof that may be utilised to prosecute and punish criminals. It may even deter certain offenders from committing more terrible offences. Additionally, forensic experts have used DNA typing techniques to re-examine ancient cases previously closed due to a lack of evidence.

## Introduction and background

The Latin term Forensis, which means “of or before the forum”, is the origin of the word “forensic”. The phrase's roots may be found in the Roman era when a criminal case, which was heard in public, was won by the person who made the most convincing arguments and delivered them with the most weight. The use of scientific knowledge to address legal issues in criminal and civil cases is known as forensic science [1]. The term “forensic” implies that something is “referred to, or employed in law court”. Forensic knowledge and its several contentious subfields are usually referred to as “forensics” or other similar abbreviations [2]. Forensic science, digital proof investigation, fingerprint proficiency, dentistry/odontology, nursing, pathology, toxicology, and papers under scrutiny are a few of the many disciplines that make up criminal science. Criminalists are committed to locating, identifying, and reassembling physical evidence using logic, reason, and scientific analysis [3].

The study of human genetic variations has enhanced forensic genetics. Blood and body fluid as physical evidence were individually recognised using common genomic markers such as the human leukocyte antigen (HLA) system, serum/plasma proteins, red cell isozymes, blood type antigens, and polymorphisms of haemoglobin [4]. Alec Jeffreys’ finding of hypervariable polymorphisms, also known as variable nucleotide tandem repeats (VNTR) or minisatellites, made it possible to employ multilocus probes for DNA fingerprinting [5]. Only monozygotic twins may have shared DNA fingerprints due to the low likelihood of matching. VNTRs are the foundation for the restriction fragment length polymorphism (RFLP) method. Single locus probes (SLP), which assess several minisatellite species under more rigorous conditions, were developed because simple pattern matches are less sensitive, technically challenging, and require microgram amounts of high molecular weight DNA. Several SLPs can be used in DNA typing to gather sufficient data to show the sample’s distinctive DNA. Techniques based on the polymerase chain reaction (PCR) that analyse the minisatellite repeats, such as the amplified fragment length polymorphism (AFLP) approach, have been developed to solve the challenges of quality and quantity in forensic evidence and the time needed for SLP analysis. Short tandem repeats (STR) or microsatellites of short repeating patterns packed in small fragments are more suited for PCR [6]. However, when there is a potential that the template DNA has been tampered with, shorter amplicons are also feasible. The focus of this study is STR profiling, which has emerged as the industry standard for forensic DNA typing. Because STRs with 3-4 bp repeats are so polymorphic, multiplexing or profiling at 4-6 loci may further reduce matching between unrelated people. In the review, it is emphasised how crucial STR indicators are to the system of criminal justice [7].

Applications of DNA testing and DNA profiling in India are used for both civil and criminal purposes in modern culture. DNA profiling is widely employed in criminal cases, notably those involving corroborative evidence and Disaster Victim Identification (DVI). It is also used in civil cases, such as those involving kinship and biological parentage [8].

## Review

Methodology

This review’s thorough electronic literature search used Google Scholar, Science Direct, PubMed, and Google Search. The terms “Forensic DNA typing”, “STR typing”, “DNA database”, “DNA probe” and “Restriction Fragment Length Polymorphism” were used alone as well as in combinations. All relevant studies were well-thought-out, including reviews, meta-analyses, organisational submissions, and original research. Although earlier, highly regarded publications were still regularly referenced, the most recent studies were given precedence.

An overview of DNA

Researchers debated which molecule held the blueprint for life. Most scientists thought DNA was an overly primary chemical to serve such an essential purpose. Instead, researchers hypothesised that proteins’ increased complexity and variety of forms boosted their capacity to do this vital task. By 1952, thanks to Alfred Hershey and Martha Chase’s ground-breaking discoveries, it was apparent how important DNA was as the genetic material [9]. The double helix configuration of DNA, which permits the transmission of biological data from one generation to the next, was discovered as a result of research by James Watson, Francis Crick, Maurice Wilkins, and Rosalind Franklin on X-ray diffraction patterns.

Every human cell has DNA, especially the nuclear DNA in the nucleus of the cell, but some DNA is also present in the mitochondria called mitochondrial DNA. DNA and its instructions are transferred from parents to children [10]. Like fingerprints, each person has a unique DNA profile that does not change over a lifetime. Except for homozygous twins, each person’s genetic makeup is distinct and follows them everywhere. This trait is used in DNA testing, also known as DNA typing [11].

The human “genome” has undergone extensive sequencing and has been entirely studied, if not construed. Less than 25,000 genes (which code for proteins) are found within the 3 billion bases of the human genome, which is spread throughout 23 chromosomes and accounts for less than 5% of the genome’s overall length [12]. The Human Genome Project has shown that repeated sequences may be found in the genome’s non-coding sections. While multilocus probes, or short tandem repeats, are dispersed across the human genomic sequence, single-locus satellites are restricted to a single human chromosomal location [13].

Repetitive DNA sequences

Within the eukaryotic genome, repetitive DNA sequences can be slightly or very repetitive. They can also be tandemly organised or scattered. Highly repeated tandem sequences comprise satellite DNAs, minisatellites, and microsatellites (VNTRs). Repeated DNA fragments’ lower density (satellite) portion is referred to as a satellite after density gradient centrifugation. Single-locus spacecraft, known as minisatellites, differ from microsatellites in design and intended use. They are constrained to particular regions of the human genome [14]. Their varied array of core repeat motifs ranges from 10 bp to 100 bp and spans 1 kb to 15 kb. A microsatellite, also known as an STR, is a homogeneous array of repeat motifs with a repetition size of less than or equivalent to 1 kb and a width of 2 bps to 6 bps. Variations also influence polymorphisms in loci in the number of repeat units. The mutation rate in the variable nucleotide tandem repeats domain is 10-100,000 times larger than the average rate in other genetic regions. Repeat units can grow or shrink due to strand slippage and rough crossings during DNA replication. Digital DNA typing’s fundamental premise is that people within a population differ in terms of the number of repeats and the arrangement of repeat types. Independent assortment also leads to allelic variations in an individual’s autosomal repeat sites [15]. Although many forensic investigations focused on minisatellite polymorphisms, STR markers were a preferred tool among researchers because of their accessibility and compatibility with PCR.

Short DNA sequences, called short tandem repeats repeated numerous times (2-6 bp), make up around 3% of human genetics. The number of repetition units varies amongst people when assessed for identifying purposes, offering a high power of discrimination. STRs are typically thought to have no role in controlling gene expression since they are non-coding. Nevertheless, there is mounting proof that non-coding DNA sequences, like short tandem repeats, may influence genes in several ways and impact phenotype.

DNA evidence and forensic science

According to Locard’s exchange principle, every interaction leaves a trail, forming criminal forensic inquiry’s basis. It is feasible to identify the malfunctioning gene by analysing the location of the RFLP in DNA if it is inside or even close to the locus of a gene that causes a specific illness. The cellular DNA of the subject is separated and subjected to restriction enzyme processing, and then electrophoresis is used to separate the DNA fragments acquired. It is possible to compare the RFLP patterns of the illness suspects with those of healthy persons (preferably with relatives in the same family). This method may identify if a person carries the illness gene and the marker RFLP. RFLPs are capable of detecting single gene-based illnesses with 95% accuracy. This claim states that the material is transferred anytime two things come into contact, and a trace is left behind. Since the victims’ bones are periodically mixed up with other evidence or severely fragmented, the conventional identification approach based on the anthropological and physical features of the target is ineffectual and inconclusive [16]. However, when there are many pieces of proof, DNA typing - the gold standard in forensic case resolution - accurately identifying victims and suspects in particular cases continues to be a helpful method. According to assertions that human DNA is 99.9% identical with only 0.1 percent variance, one in 594.1 trillion people who are not blood-related will have the same DNA sequence. Given this, it should be evident that DNA testing has vindicated the innocent and established the guilt of the guilty [17]. Studies show that in some countries, more than half of the DNA typing samples are obtained from touching things. This is primarily due to the discovery that DNA may be found in biological material that cannot be seen without contacting a surface with one's hands. A single interaction incident can simultaneously involve direct/primary and indirect/secondary transmission incidents [18]. A self-DNA put within the handprint may be considered a preliminary deposit instead of a non-self-capability, characterised as a secondary deposit. Other frequent biological specimens for DNA extrication and examination include saliva, blood, nails, and tooth filaments.

Dr. Alec J. Jeffreys, a genetics professor at the University of Leicester, used DNA fingerprinting for the first time in forensic science when British forces asked him to profile an accused in the rape and murder of 15-year-old Dawn Ashworth in Leicestershire [19]. Dr. Jeffreys had already made the case that distinct DNA sequences may be utilised to distinguish between individuals before the occurrence. The primary suspect in the case, Richard Buckland, even admitted to murdering Ashworth [20]. After analysing samples from Buckland, and 1983 and 1986 murder sites using DNA typing, Dr. Jeffreys found comparable DNA at both crime scenes. Nevertheless, the obtained DNA lacked Buckland's DNA profile. To discover the actual offender, the law enforcement agency utilised a genetic search to gather blood and saliva specimens from over 4,000 males in the Leicestershire area between the ages of 17 and 34 [21]. However, no reliable match was found. However, a guy was overheard saying that he had provided bogus samples and had been paid to pose as someone else [22]. The DNA dragnet was attempting to catch up with Colin Pitchfork. The DNA of Pitchfork was examined, and it matched samples from the crime scene. On September 19, 1987, Pitchfork was taken into custody. In January of the following year, he was finally determined guilty and sentenced to life in prison. He was the first killer to have DNA evidence to support his conviction. Thanks to DNA evidence that linked his genetic code to semen traces discovered on the victim, Tommy Lee Andrews was convicted of rape in the United States the same year as the Pitchfork conviction (1987).

The double murder trial of Timothy Wilson Spencer v. the State of Virginia in 1994 and Glen Dale Woodal v. the State of West Virginia in 1992 are two notable instances that advanced the use of DNA evidence [23]. In the Woodal case, DNA evidence resulted in the defendant's discharge, while in the Spencer case, it led to the defendant's conviction and death sentence. Since the Pitchfork case in 1987, DNA analysis techniques have seen considerable scientific development. To amplify the implementation of DNA profiles in investigations, the United Kingdom National DNA Database was significantly created in 1995 [24]. Today, forensic DNA analysis is used in some capacity in the majority of the world's countries. Whose DNA is it? What body fluid did it originate from? How did it get there? And were the findings reported accurately and unbiasedly? These are the crucial questions that a forensic DNA expert must ask.

The admissibility of DNA evidence has recently caused a great deal of debate in courts worldwide. The science behind it is that it is reliable, repeatable, and accurate since it is based on methods and equipment that are legal to develop and analyse DNA profiles [25]. The National Research Council's (NRC) 1996 study on DNA proof said that the admission of correctly gathered and analysed DNA data “should not be in question.” According to the paper, “the status of the technology for profiling and the approaches for calculating occurrences and associated information have advanced to this stage.” Table 1 shows the differences between DNA fingerprinting and DNA profiling [26].

**Table 1:** Differences between DNA fingerprinting and DNA profiling DNA - Deoxyribonucleic Acid; STR - Short Tandem Repeats; VNTR - Variable Number of Tandem Repeats; AFLP - Amplified Fragment Length Polymorphism; RFLP - Restriction Fragment Length Polymorphism; PCR - Polymerase Chain Reaction.

DNA FINGERPRINTING	DNA PROFILING
The use of DNA analysis to identify people.	The examination of a person's DNA traits for forensic studies.
A molecular genetic technique that identifies people based on their distinctive DNA sequences	A forensic procedure crucial to parentage testing and criminal investigations.
Focuses on VNTRs, which include both micro- and mini-satellites.	STRs, which are microsatellites, are the main emphasis.
Techniques used - RFLP, AFLP and PCR	Techniques used - PCR
A lengthy process with several phases.	A straightforward technique that is automatable.

Classification of tandem repeats

I. STRs (Short tandem repeats or micro-satellites): Short tandem repeats are sequences where the repeating unit has a base pair length of 1-6 bps.

II. VNTRs (Variable number tandem repeats or mini-satellites): Variable number tandem repeats are sequences where the repeating unit has a length of 7 to 100 bps (base pairs).

III. Satellite DNA: The sequence is referred to be satellite DNA if the repeating unit is between 100 and several thousand base pairs in length.

DNA typing process

**Figure 1:**
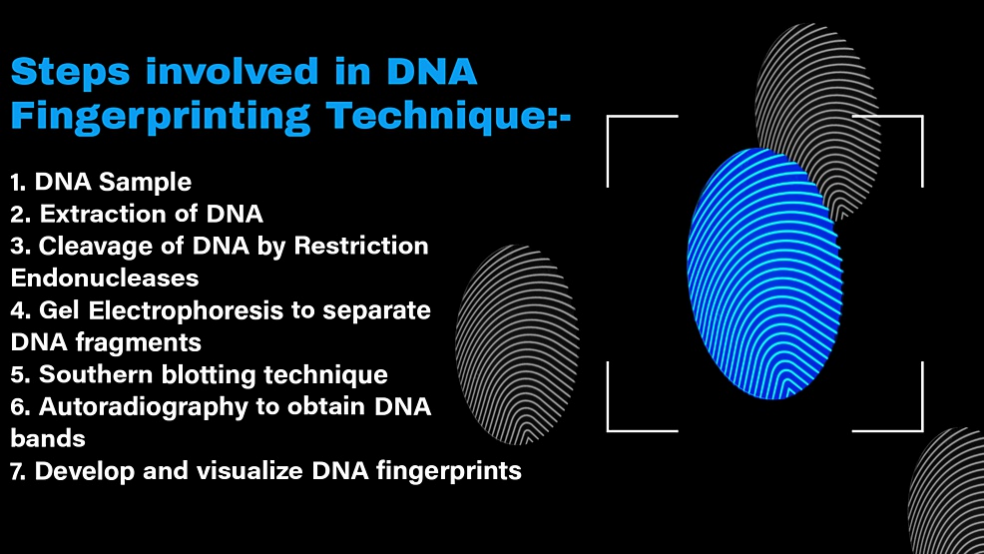
Steps involved in DNA fingerprinting technique DNA - Deoxyribonucleic Acid This is a self-made figure by the author.

The technique begins with a reference sample, which is a DNA sample from a human. The ideal method for acquiring a reference specimen is to use a buccal swab since it decreases the occurrence of infection [27]. Alternative approaches may need to be used to get a specimen of blood, saliva, semen, or another acceptable fluid or tissue from a person or previously kept samples if this is not possible (for example, if a court order is necessary but not always attainable) (e.g., banked sperm or biopsy tissue). The analysis of a reference specimen is then used to generate the person's DNA profile [28,29]. The DNA profile is then contrasted with a separate specimen to see whether there is a genomic equivalence. Figure 1 shows the steps involved in DNA fingerprinting technique.

STR typing

Years ago, restricted fragment length polymorphism, a DNA profiling technique, was abandoned in favour of PCR procedures, which amplified DNA. After numerous PCR-based procedures were improved upon and modified, STRs were accepted by the forensic community [30]. Due to the expense of infrastructure, training, databases, and certification, STR typing will continue to be the technique of selection for most forensic laboratories, even if additional DNA markers are utilised. Microsatellite loci have been discovered, recognized, and established to be remarkably prevalent in the human genetics [31]. When commercial kits were created, the enormous abundance and polymorphism characteristics of STR loci were taken into consideration. Since then, forensic laboratories have continued to employ this method as the most popular and current industry standard for using human identification.

The recognised DNA typing approach is followed by STR typing analysis [32]. On the other hand, the STR typing method is based on the standard operating procedures (SOPs) supplied by the manufacturers of the commercial kit for use in forensic laboratories. The following steps are taken for STR typing in the conventional order for DNA typing: DNA extraction is performed to decide the amount of DNA present, amplify STR loci, separate PCR amplicons on a genetic analyser, examine the resultant data using bioinformatics, and compare the data from one sample to directories with previously generated short tandem repeats sets [33]. The size and colour of these amplicons may be used to differentiate STR loci, which are repetitive DNA sequences with variable repetition counts produced using primers with distinct fluorophores. Another crucial step in analysing STR loci is identifying the invariant flanking areas surrounding the repeats [34]. If the adjacent sequences are known, the repeat region may be amplified using PCR primers. Typically, there are two methods to calculate short tandem repeats. These comprise using molecular science isolation techniques or searching DNA sequence directories like Combined DNA Indexed System or GenBank for areas above vicinal repeat units [35].

Resolution of cases using STR typing

These include using molecular science isolation techniques or searching DNA sequence directories like Combined DNA Indexed System or GenBank for areas above six vicinal repeat units [36]. Forensic experts use DNA typing to connect the case to the individual who has been found by comparing the trailed proof to the suspect. When there is no obvious suspect, DNA samples taken at the incident should be verified with DNA profiles stored on a national DNA directory to check if there is an equivalence or hit between the proof before you and a profile in the directory [37].

Discussion

DNA profiling offers uncontestable proof of erroneous convictions, invaluable connections to genuine criminals, and the potential to dissuade certain offenders from committing even more horrible crimes worldwide [38]. Additionally, forensic experts have re-examined earlier cases that were ruled closed owing to a lack of evidence using DNA typing techniques. One of these well-known examples of how a DNA typing technique helped free people who had been unfairly detained is the Innocent Project. The 1992 founding of the American non-profit saw the exoneration of 272 people, including 17 people who were on death row [39].

Restrictions fragment length polymorphism, initially established in the middle of the 1980s, was the first DNA typing method. DNA was typed using the RFLP method, which comprised central elements of sequences made up of 30-100 repetitions (variable number tandem repeats). A significant amount of intact genomic DNA is needed for DNA characterization using the restriction fragment length polymorphism technique (20 to 30 mg). However, the biological samples brought into a lab for forensic science are frequently subjected to environmental abuse, and sometimes only trace amounts of DNA may be extracted. As a result, the restriction fragment length polymorphism technique was often inapplicable.

Short tandem repeats typing is the present DNA typing technique. With this technique, multiple loci made up of nucleotide repeats ranging from 80 to 400 base pairs may be co-amplified, and automated DNA fragment examination can produce the findings on the same day. This technology is better than the restriction fragment length polymorphism approach since it can test degraded materials and only needs a small quantity of DNA (0.5 to 1 ng). For this reason, the courts are enabling cases to be reopened if information from evidence or technology not accessible at the initial trial (such as DNA) may exonerate a wrongfully convicted person.

Numerous violent crimes, including killings and assaults, have resulted in convictions thanks to DNA analysis. Additionally, it has resulted in the exoneration and release of those who had previously been convicted and the elimination of suspects. DNA can concentrate an investigation, likely to expedite trials and result in guilty pleas. Some criminals could be discouraged from committing significant offences as a result. The criminal justice system will save money over time as a result of using forensic DNA evidence more frequently. DNA testing is a scientific process that offers valuable evidence to both the prosecution and the defence. Due to DNA analysis, one-third of the first suspects are exonerated before trial, saving the taxpayers money, the court's time, and most importantly, the innocent person's freedom.

Regardless of how recently a crime was committed, these examples show the value of DNA databases and profiling as investigative tools [40]. By creating national and regional DNA data banks for each state, or at least two states, law enforcement authorities in India have boosted their use of DNA data banking in recent years. Long-term budgetary benefits for the criminal justice system will also come from the continuous use of forensic DNA evidence. It is essential to establish a national DNA database and put in place legal and administrative frameworks that would enable and enhance the practice of forensic genetics, mainly DNA typing, in developing countries like Nigeria [41].

## Conclusions

The current research comprehensively demonstrates the usage of short tandem repeats in criminal investigations through references to key case reports and historical incidents, given that STRs are crucial to DNA typing. We consider this evaluation a teaching tool for aspiring forensic professionals in developing countries where forensic research in DNA typing is still in its infancy.

Situations can be explicitly handled, and paternity can be established via DNA fingerprinting. Both the pursuit of the criminal and the exoneration of the innocent use DNA profiling as a tool. DNA evidence is unaffected by falsification of other types of evidence or hostile witnesses, as is frequently the case in the administration of justice. It is not affected by the course of time, nor does it alter. Thus, DNA evidence reveals the truth since it never tells lies. We will undoubtedly continue to witness a rise in the use of these tests and the accessibility of comprehensive genetic services to the general population as DNA sequencing and analytical technology progresses.
